# Stress hyperglycemia as first sign of asymptomatic type 1 diabetes: an instructive case

**DOI:** 10.1186/s12887-021-02811-z

**Published:** 2021-08-06

**Authors:** Wei-De Wang, Chun-Hao Chu, Chiung-Hsi Tien, Shuo-Yu Wang, Shih-Yao Liu, Chien-Ming Lin

**Affiliations:** 1 Department of Pediatrics, Tri-Service General Hospital, National Defense Medical Center, No. 325, Cheng-Kung Road, Section 2, Neihu 114, Taipei, Taiwan, Republic of China; 2 Department of Pediatrics, Kaohsiung Armed Forces General Hospital Zuoying Branch, Kaohsiung, Taiwan; 3 Department of Pediatrics, Kaohsiung Medical University Hospital, Kaohsiung Medical University, Kaohsiung, Taiwan; 4 Department of Pediatrics, National Taiwan University Hospital and College of Medicine, National Taiwan University, Taipei, Taiwan

**Keywords:** Children, Hyperglycemia, Stress, Type 1 diabetes mellitus

## Abstract

**Background:**

Stress hyperglycemia (SH) is considered a transient manifestation and routine diagnostic evaluation was thought to be unnecessary due to the lack of definite correlation with diabetes mellitus (DM). Although SH was usually benign and long-term treatment was superfluous, it might be the first sign of insulinopenic status such as type 1 DM (T1DM).

**Case presentation:**

We reported a boy with acute asthma attack presented incidentally with high blood glucose levels exceeding 300 mg/dL and obvious glycemic variability. A prolonged hyperglycemic duration of more than 48 h was also noticed. To elucidate his unique situation, glucagon test and insulin autoantibody survey were done which showed insulinopenia with positive anti-insulin antibody and glutamic acid decarboxylase antibody despite the absence of overt DM symptoms and signs.

**Conclusions:**

This case highlights that SH might be a prodromal presentation in T1DM children, especially when accompanied simultaneously with extreme hyperglycemia, apparent glucose variability, as well as prolonged hyperglycemic duration.

## Background

Stress hyperglycemia (SH) is a common clinical manifestation in children with acute illness. It is caused by the increased levels of cortisol, catecholamine and proinflammatory cytokines (TNF-α, IL-1 and IL-6) mediated by the hypothalamic-pituitary-adrenal axis and the sympathoadrenal system. Since the physiological response of SH increases the glucose uptake of the brain and immune system at a time of stress which in turn enhances the chances of survival, the meticulous investigation and aggressive management to SH were thought to be unnecessary in clinical practice [1]. The prevalence of hyperglycemia, defined as glucose level≧150 mg/dl, has been reported to be ranged from 3.8 to 4.9% children in the emergency department [2–4]. On the other hand, among ill children in intensive care unit (ICU), SH accounted for 36.6 and 44.5% of them at the initial time and within 24 h of the admission, respectively [5]. Because the SH seems associated with the severity of underlying disease, it was usually regarded as a benign phenomenon in acute illness. However, whether the coexistence of other pathogenic etiology could mask or aggravate SH has never been emphasized in the literature.

Previous studies showed no obvious association between SH and type 1 diabetes mellitus (T1DM) [2, 3, 6–10]; therefore, the routine workup for children with SH to confirm T1DM is not recommended while there is no overt signs and/or symptoms of DM. To add a new dimension to the physiological role of SH, we herein reported an asthmatic boy having unique SH with manifestations of extreme hyperglycemia, glucose variability (GV), and prolonged hyperglycemic duration, finally confirmed as T1DM. This instructive case highlights an unusual pattern of SH with possible hidden pathogenesis and further investigation should be considered for precise early diagnosis to prevent subsequent diabetic ketoacidosis.

## Case presentation

An 8-year-2-month old Taiwanese boy presented with dyspnea for 1 day. He had no preceding symptoms such as polyphagia, polydipsia, polyuria, or body weight loss during this episode. His past medical history is notable for bronchial asthma and allergic rhinitis without using regular medication. Family history revealed no T1DM or T2DM in family members. Two days before admission, he was noted to have runny nose, productive cough and fever. He was treated with antitussives and antipyretics at a local clinic initially. Because dyspnea along with decreased oral intake was noticed 1 day later, he was sent to primary healthcare center where inhaled terbutaline sulfate and intramuscular dexamethasone were given in consideration of acute exacerbation of bronchial asthma (AEBA). After treatment, laboratory tests showed no acidosis or alkalosis; however, hyperglycemia was noticed (Table 1). Owing to persistent dyspnea, he was referred to our hospital.

**Table 1:** Laboratory data of patient

**At primary healthcare center**					
**Biochemistry**			**Venous blood gas analysis**		
Glucose	381 mg/dL	(70–100)	pH	7.391	(7.32–7.43)
Na^+^	133 mmol/L	(136–145)	PvCO_2_	31 mmHg	(38–49)
K^+^	4.3 mmol/L	(3.5–5.1)	PvO_2_	53.4 mmHg	(30–50)
Ketone	0.7 mmol/L	(< 0.6)	HCO_3_	19.0 mmol/L	(22–29)
CRP	0.5 mg/dL	(< 0.8)	BE	−4.4 mmol/L	(− 4 − + 2)
**On admission**					
BH 127 cm (25-50th percentile), BW 23 kg (15th percentile), BT 37.1 °C, HR 116 beats/min RR 32 breaths/min, BP 115/64 mmHg, SpO_2_ 99% (nasal cannula with O_2_ flow rate 3 L/min)					
**Blood cell count**			**Urinalysis**		
WBC	10,690/μL	(4000–12,000)	pH	5.0	(4.5–8.0)
Hb	13 g/dL	(11.5–14.5)	Glucose	4+	(negative)
Plt	25.8 × 10^4^ /μL	(15 × 10^4^–40 × 10^4^)	Ketone body	2+	(negative)
Neutrophil	91%	(54–62)	Occult blood	–	(negative)
Lymphocyte	6.7%	(25–33)	Strip WBC	–	(negative)
**Biochemistry**			**Arterial blood gas analysis**		
Glucose	373 mg/dL	(70–100)	pH	7.318	(7.35–7.45)
Na^+^	134 mmol/L	(136–145)	PaCO_2_	49 mmHg	(35–45)
K^+^	4.1 mmol/L	(3.5–5.1)	PaO_2_	67.6 mmHg	(75–100)
Cl^−^	98 mmol/L	(98–107)	HCO_3_	24.8 mmol/L	(21–28)
AST	14 IU/L	(< 50)	BE	−1.8 mmol/L	(−4 − + 2)
ALT	8 IU/L	(< 45)	SaO_2_	92%	(> 95)
BUN	12 mg/dL	(5–18)			
Cre	0.5 mg/dL	(0.3–0.7)			
Ketone	0.9 mmol/L	(< 0.6)			
HbA1C	7.7%	(4.0–5.7)			
**Islet autoantibodies**			**Glucagon stimulation test**		
Insulin Ab	5.7% B/T	(< 5.5)	Time	C-peptide	
Anti-GAD	7.5 U/mL	(< 1.0)	0 min	0.43 ng/ml	(> 0.5)
Anti-TPO	15.2 U/mL	(< 35)	6 min	1.16 ng/ml	(> 1.8)
Anti-TG	5.0 U/mL	(< 20)			

*Ab* antibody, *Anti-GAD* anti-glutamic acid decarboxylase antibody, *Anti-TPO* anti-thyroid peroxidase antibody, *Anti-TG* anti-thyroglobulin antibody, *AST* aspartate aminotransferase, *ALT* alanine aminotransferase, *BE* base excess, *BH* body height, *BP* blood pressure, *BT* body temperature, *BUN* blood urea nitrogen, *BW* body weight, *Cl*^*-*^ chloride, *Cre* creatinine, *CRP* C-reactive protein, *Hb* hemoglobin, *HbA1C* hemoglobin A1c, *HR* heart rate, *K*^*+*^ potassium, *Na*^*+*^ sodium, *Plt* platelet, *RR* respiratory rate, *SaO*_*2*_ arterial oxygen saturation, *SpO*_*2*_ O_2_ saturation by pulse oximetry, *WBC* white blood cells

On examination, the patient was alert but distressed. He had normal skin turgor and no dehydrated mucous membranes. Use of accessory muscle and bilateral diffuse wheezing were noticed. There was no acanthosis nigricans over posterior neck or axillae. The thyroid was non-palpable. Laboratory tests showed hyperglycemia, ketonuria and ketonemia (Table 1). Chest radiograph revealed bilateral pulmonary infiltration. Under impression of AEBA, he was admitted to pediatric ICU (PICU).

**Fig. 1:**
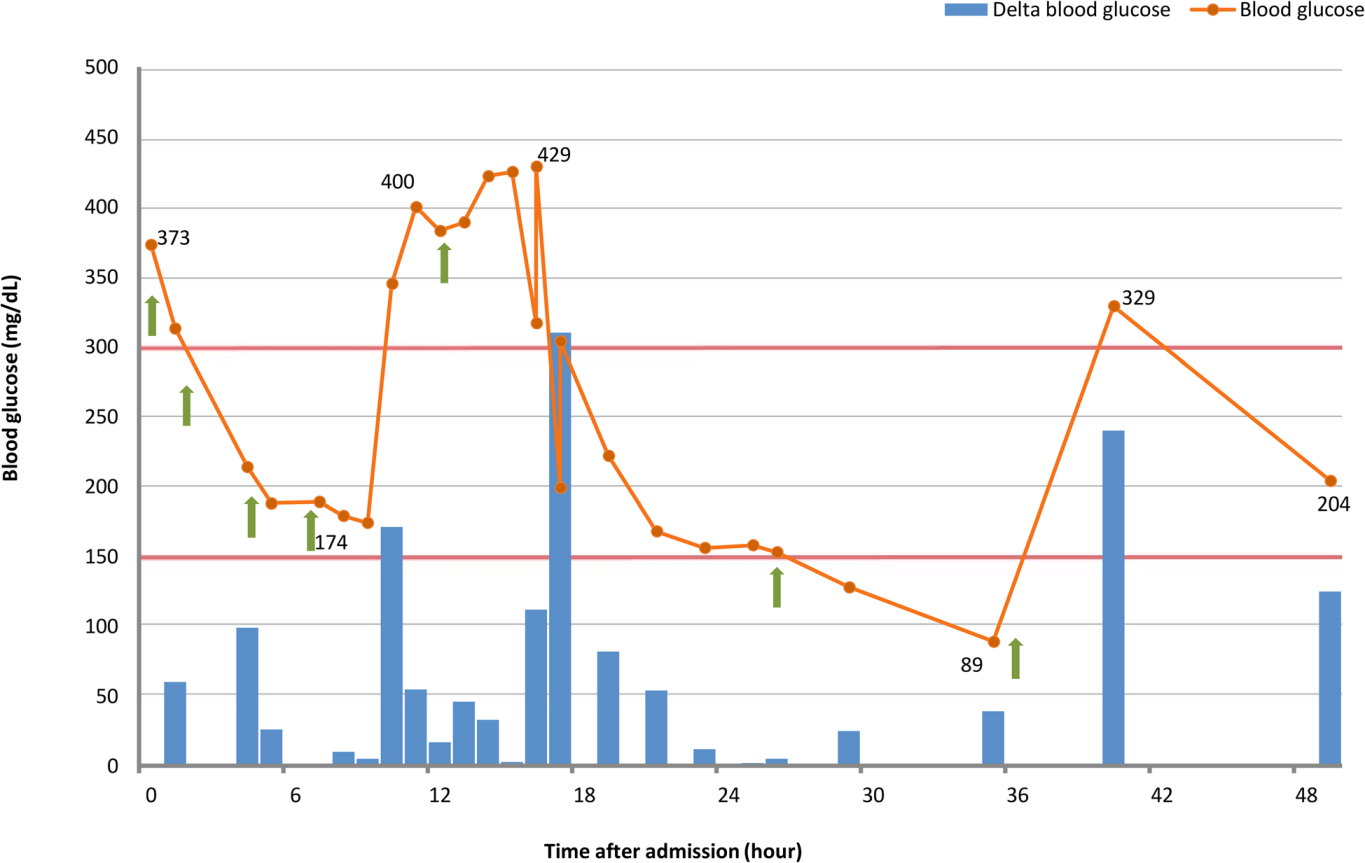
Blood glucose monitoring of patient after admission. Blood glucose concentration (orange line and dots) measured by regular fingerstick testing over two consecutive days after admission. Delta blood glucose (blue bars) was defined as change in blood glucose values between two adjacent time points, which also represents the trend of glycemic variability over time. Green arrows indicate the use of beta-2 agonists or corticosteroids

On observing hyperglycemia, exclusively half-normal saline was infused on the first day of hospitalization. Inhaled beta-2 agonists and intravenous corticosteroids were also given. Nevertheless, blood glucose monitoring disclosed fluctuating hyperglycemia. Although this SH might result from AEBA per se and/or medication, his baseline blood glucose levels were inexplicably higher than 150 mg/dL within the first 24 h and frequently rose above 300 mg/dL or even 400 mg/dL (Fig. 1). The delta blood glucose levels (△BG) could reach 150 to 300 mg/dL in a single hour, suggesting extremely high GV. Moreover, blood glucose exceeding 150 mg/dL could be detected even at 48 h after admission, indicating prolonged hyperglycemic duration. To elucidate this unusual fluctuation of glucose values, further investigations were done which showed increased hemoglobin A1c (HbA1c), positive islet autoantibodies, and insulinopenia in glucagon test (Table 1). Finally, he was diagnosed as T1DM despite the absence of classical DM symptoms.

## Discussion and conclusions

SH is regarded as benign and transient hyperglycemia during acute stress. Accumulating studies have demonstrated that SH was unrelated to T1DM, thus rendering routine confirmatory investigation unnecessary [2, 3, 6–10] (Table 2). Furthermore, extreme SH (ESH), glucose levels ≥300 mg/dL, was rarely seen in children; and it was also unrelated to subsequent DM [10]. Crucially, previous studies showed that only 13% of ESH patients had ketonuria [10]. In view of this, ketonuria in our young boy might be taken as a feature of glucose dysregulation in that ketone body forms rapidly in insufficient insulin environment. Therefore, the current latent T1DM case is an exceptional didactic experience worthy of attention.

**Table 2:** Summary of studies on the relationship between stress hyperglycemia and diabetes mellitus

Study	Numbers/Age/Country	Analysis and Results	SH association with DM	Strength	Limitation
Our case	A boy with AEBA 8 yrs Taiwan	Case report Analyze the SH pattern by regular stick blood glucose test Evaluate HbA1C, glucagon test, insulin Ab, urinalysis	Subclinical T1DM is associated with ESH (> 300 mg/dL), high glucose variability (△BG > 150 mg/dL), prolonged hyperglycemic duration (> 48 h) and ketonuria	Offer unique hints of lactent DM during SH Delicate evaluation of T1DM (HbA1C, glucagon test, insulin Ab) Evaluate glucose variability	Case report
Gupta et al. 1997 [2]	758 ill children 1 m/o to 6 yrs. Delhi, India	Cross-sectional study SH defined as ≥150 mg/dL SH prevalence: 4.7% (36/758) All SH resolved within 24 h from admission No significant association between SH and severity of illness	OGTT were performed in 31 SH subjects and all revealed normal results Urinalysis were performed in 30 SH subjects and no ketonuria was found	Large population-based Using OGTT to confirm DM	Exclude subjects with beta 2 agonist and steroid Tx Without insulin Ab evaluation
Bhisitkul et al. 1994 [3]	926 ill children 3 days to 21 yrs. Norfolk,Virginia	Cross-sectional and longitudinal study SH defined as ≥150 mg/dL SH prevalence: 3.8% (35/926) SH is associated with severity such as high fever, ICU admission, and intravenous hydration	No SH subjects were diagnosed with DM	Large population-based Cross-sectional and longitudinal (mean f/u 4 to 9 m/o)	Exclude subjects with beta 2 agonist and steroid Tx
Shehadeh et al. 1997 [6]	36 ill children 1 to 17 yrs. Haifa, Israel	Longitudinal study SH defined as ≥150 mg/dL No subject was diagnosed with DM	SH is a low risk factor of T1DM	Longitudinal analysis (mean f/u 3.2 yrs) Evaluation of T1DM (serum Ab, IVGTT)	Small population-based No asthmatic subject No control group
Herskowitz-Dumont et al. 1993 [7]	63 children with transient hyperglycemia 1 to 18 yrs. Boston, Massachusetts	Longitudinal study Hyperglycemia defined as ≥150 mg/dL 19 healthy subjects; 44 with illness (11 asthmatic subjects) 32% (6/19) healthy subjects and 2.3% (1/44) ill subjects were diagnosed with T1DM	Transient hyperglycemia is a high risk to develop T1DM in healthy subjects, but a low risk in ill subjects No asthmatic subject developed T1DM	Longitudinal analysis (mean f/u 7 yrs) Evaluation of T1DM (serum Ab, IVGTT) Enrolled asthmatic subjects	Small population-based No control group
Eshraghi et al. 2014 [8]	50 children with history of SH Average 9.8 yrs. Babol, Iran	Retrospective cohort study SH defined as ≥200 mg/dL No subjects were diagnosed with DM Insulin resistance in 16% (8/50) subjects	SH is a low risk factor of T1DM but may be related to Insulin resistance	Evaluation of T1DM (serum Ab, OGTT)	Small population-based Exclude subjects with beta 2 agonist and steroid Tx
Bhisitkul et al. 1996 [9]	30 ill children with SH, 30 ill children without SH, 30 healthy subjects 4 weeks to 12.4 yrs. Norfolk, Virginia	Case-control and longitudinal study SH defined as ≥150 mg/dL No subjects were diagnosed with DM	SH is a low risk factor of T1DM	Longitudinal analysis (mean f/u 31 to 36 m/o) Evaluation of T1DM (serum Ab) Compared with two control goups	Exclude subjects with beta 2 agonist and steroid Tx No asthmatic subject
Weiss et al. 2010 [10]	55,120 ill children including 72 ESH subjects Average 8.8 yrs. (ESH subject) Boston, Massachusetts	Retrospective cohort study ESH defined as ≥300 mg/dL ESH prevalence: 0.13% (72/55120) Asthmatic children account for 31% (22/72) ESH subjects ESH is associated with increased mortality and severity of illness Only one subject was diagnosed with steroid induced DM	No asthmatic subject developed T1DM or T2DM No association between ESH and DM Urinalysis was performed in 53% (38/72) ESH subjects and ketonuria was found in 13% of them	Large population-based Enrolled asthmatic subjects Include subjects with β2 agonist and steroid Tx	No control group
Jin-Sun Chang et al. 2013 [11]	Mice with diabetes (animal model)	Animal study Analyze the SH pattern of diabetic mice under predator stress (cat) Distinct SH pattern among different types of DM	T1DM exhibited the “fast & slow” pattern during SH	Describe glucose fluctuation	Not human model Psychiatric stress may be different from physiological stress

Abbreviation: *Ab* autoantibody, *AEBA* acute exacerbation of bronchial asthma, *DM* diabetes mellitus, *ESH* extreme stress hyperglycemia, *f/u* follow up, *IVGTT* intravenous glucose tolerance test, *MetS* metabolic syndrome, *m/o* month, *OGTT* oral glucose tolerance test, *SH* stress hyperglycemia, *T1DM* type 1 diabetes mellitus, *T2DM* type 2 diabetes mellitus, *Tx* treatment, *yrs.* years

In addition to AEBA per se, beta-2 agonists and corticosteroids also increase blood glucose mediated by promoting gluconeogenesis. Accordingly, previous studies regarding SH almost excluded beta-2 agonists and corticosteroids intervention [2, 3, 8, 9] (Table 2); therefore, asthmatic children with underlying DM might be excluded and then underestimated. Interestingly, asthma and T1DM are both immune-mediated disease but their association was not fully clarified. It was reported that children with asthma increase the risk of subsequent T1DM development by 41% when compared to normal population [12]. To date, there were only two studies on a total of 33 asthmatic children exploring the relationship between transient hyperglycemia and asthma, but none of the subjects was diagnosed to have DM eventually [7, 10].

Likewise, in our patient, bronchodilator and steroid might contribute to hyperglycemia and aggravate the severity of SH; hence, drug effect on glucose metabolism should be taken into consideration. Burgess C. et al. reported that only high doses of inhaled beta-2 agonist can significantly increase blood glucose to the peak level of 133 mg/dL after 5 h of treatment in mild-to-moderate asthma subjects [13]. Another study showed that asthmatic patients had a peak value of glucose 293 mg/dL after 4 h of combined treatment with beta-2 agonist and corticosteroid [14]. In the present case, extreme values of blood glucose were inconsistent with the time point of beta-2 agonists and corticosteroids intervention (Fig. 1). Moreover, the inexplicably higher glucose concentration (≥ 300 mg/dL) than those in previous studies, which together supported that dysglycemia of our case might be caused not only by acute stress and drug effect but also by underlying insulinopenia. In addition to peak blood glucose, the duration of SH could be another crucial clue for early detection of latent DM. It has been reported that 67% of ESH subjects normalized their blood glucose levels below 150 mg/dL within 48 h and none of them was subsequently diagnosed with DM [10]. Similarly, Gupta et al. also demonstrated the restoration of SH could be observed within 24 h in all enrolled children with illness and the result of oral glucose tolerance tests done for 86% participants were normal [2]. In the present case, blood glucose above 150 mg/dL was detected even at 48 h after admission (Fig. 1), indicating that this prolonged SH might be caused by latent DM.

To elucidate the pattern of glucose fluctuation under psychiatric stress, T1DM animal models under a predator stress circumstance showed the SH was characterized as “fast and slow” pattern, in which blood glucose rapidly increased and slowly decreased thereafter [11]. Although we cannot fully extrapolate the animal results to humans, blood glucose levels of the present case indeed rapidly increased from 174 mg/dl to 400 mg/dL within 2 h (9th–11th hours after admission), maintained peak levels above 400 mg/dL for 5 h (11th–15th hours after admission) and then decreased slowly to 89 mg/dL (Fig. 1), suggesting a human “fast and slow” pattern along with prolonged SH course in insulinopenic status.

Although HbA1c was a pivotal biomarker of long-term glycemic control with the usefulness to reflect the cumulative glycemic history of the preceding two to three months [15], it cannot tell GV to clinicians. It has been reported that GV was associated with diabetes complication and increased mortality rate in PICU [16, 17]. Hanefeld et al. monitored blood glucose values with continuous glucose measurement systems (CGMS) in abnormal glucose tolerance subjects and control ones and demonstrated a significantly higher amplitude of glucose excursion and standard deviation (SD) in those with abnormal glucose tolerance, suggesting higher GV [18]. Another study focusing on children with positive islet autoantibodies (high risk to future T1DM) revealed those with blood glucose above 140 mg/dL accounted for over 20% study time during a 5- to 7- day -period of CGMS monitor could be used to predict the future development of DM. Compared to patients with negative antibody, a higher SD of blood glucose was noted in those with positive islet autoantibodies [19].

Owing to unconfirmed diagnosis of DM at admission, regular stick blood glucose test was performed for our patient to evaluate his GV under stress instead of real-time CGMS. Significant change of blood glucose was noticed initially after stress exposure; however, obvious variation unexpectedly existed after the resolution of acute stress (Fig. 1). This interesting finding illuminated persistent high GV after stress might be a potent risk factor for underlying T1DM, but further study with more patients is needed to confirm our current findings.

To our knowledge, there has been no prospective study exploring the difference in SH between diabetic and nondiabetic children. This first case highlights an important but still complex relationship between SH (glucose dysregulation) and subclinical T1DM (insulinopenia). We suggest that clinicians should be aware of pathological SH, particularly when it was characterized by features such as ESH (> 300 mg/dl), high GV even after acute phase of illness, prolonged hyperglycemic duration (longer than 48 h) or ketonuria (urine ketone ≥2+), all suggesting that dysglycemia might result from latent diabetes. Then further investigations to traditionally benign SH should be conducted even there were no clinical signs or symptoms of diabetes.

## Data Availability

All the data generated and/or analyzed during this study are included in this published article.

## Abbreviations

AEBA: Acute exacerbation of bronchial asthma
CGMS: Continuous glucose measurement systems
DM: Diabetes mellitus
ESH: Extreme stress hyperglycemia
GV: Glucose variability
HbA1c: Hemoglobin A1c
ICU: Intensive care unit
PICU: Pediatric intensive care unit
SD: Standard deviation
SH: Stress hyperglycemia
T1DM: Type 1 diabetes mellitus
